# Biochemical properties of *Paracoccus denitrificans* FnrP: reactions with molecular oxygen and nitric oxide

**DOI:** 10.1007/s00775-015-1326-7

**Published:** 2016-01-20

**Authors:** Jason C. Crack, Matthew I. Hutchings, Andrew J. Thomson, Nick E. Le Brun

**Affiliations:** Centre for Molecular and Structural Biochemistry, School of Chemistry, University of East Anglia, Norwich, NR4 7TJ UK; School of Biological Sciences, University of East Anglia, Norwich, NR4 7TJ UK

**Keywords:** Fumarate–nitrate reduction regulator, Gene regulation, Iron–sulfur cluster, Oxygen, Nitric oxide

## Abstract

In *Paracoccus denitrificans*, three CRP/FNR family regulatory proteins, NarR, NnrR and FnrP, control the switch between aerobic and anaerobic (denitrification) respiration. FnrP is a [4Fe–4S] cluster-containing homologue of the archetypal O_2_ sensor FNR from *E. coli* and accordingly regulates genes encoding aerobic and anaerobic respiratory enzymes in response to O_2_, and also NO, availability. Here we show that FnrP undergoes O_2_-driven [4Fe–4S] to [2Fe–2S] cluster conversion that involves up to 2 O_2_ per cluster, with significant oxidation of released cluster sulfide to sulfane observed at higher O_2_ concentrations. The rate of the cluster reaction was found to be ~sixfold lower than that of *E. coli* FNR, suggesting that FnrP can remain transcriptionally active under microaerobic conditions. This is consistent with a role for FnrP in activating expression of the high O_2_ affinity cytochrome *c* oxidase under microaerobic conditions. Cluster conversion resulted in dissociation of the transcriptionally active FnrP dimer into monomers. Therefore, along with *E. coli* FNR, FnrP belongs to the subset of FNR proteins in which cluster type is correlated with association state. Interestingly, two key charged residues, Arg140 and Asp154, that have been shown to play key roles in the monomer–dimer equilibrium in *E. coli* FNR are not conserved in FnrP, indicating that different protomer interactions are important for this equilibrium. Finally, the FnrP [4Fe–4S] cluster is shown to undergo reaction with multiple NO molecules, resulting in iron nitrosyl species and dissociation into monomers.

**Electronic supplementary material:**

The online version of this article (doi:10.1007/s00775-015-1326-7) contains supplementary material, which is available to authorized users.

## Introduction

*Paracoccus denitrificans* is a popular model organism and one of the best studied prokaryotes with respect to respiration. It has a remarkable metabolic versatility allowing it to thrive in aerobic or anaerobic environments [1, 2]. Under anaerobic conditions nitrogen oxides can be utilized as terminal electron acceptors, in place of oxygen, and *P. denitrificans* is one of several organisms for which the denitrification pathway is well understood. It expresses four essential reductases which sequentially reduce nitrate (*nar*), nitrite (*nir*), nitric oxide (*nor*) and nitrous oxide (*nos*) to dinitrogen [3]. Optimal switching from aerobic respiration to the denitrification pathway is thus a key requirement for this flexibility, and the coordination of the denitrification enzymes is tightly controlled at the transcriptional level [4].

In *E. coli*, the fumarate and nitrate reduction (FNR) transcriptional regulator is responsible for sensing environmental levels of O_2_ and controlling the switch to anaerobic nitrate respiration [5]. FNR proteins represent a major sub-group of the cyclic-AMP receptor protein (CRP) family of bacterial transcriptional regulators, and, like CRP, consist of two distinct domains that provide DNA-binding and sensory functions [6–8]. However, unlike *E. coli*, some bacterial species possess multiple members of the FNR protein family [4, 9]; *P. denitrificans* has three major FNR paralogues which coordinate the regulation of the denitrification enzymes [4]. One of these paralogues, NarR, is a nitrate sensor involved in regulating nitrate reductase (*nar*) expression. The second, NnrR, is a heme-based nitric oxide sensor involved in the control of expression of nitrite (*nir*), nitric oxide (*nor*) and nitrous oxide reductases (*nos*) [4]. The third, FnrP, is a true orthologue of *E. coli* FNR as it regulates genes encoding aerobic and anaerobic respiratory enzymes in response to O_2_ availability [4, 10–12].

**Fig. 1:**
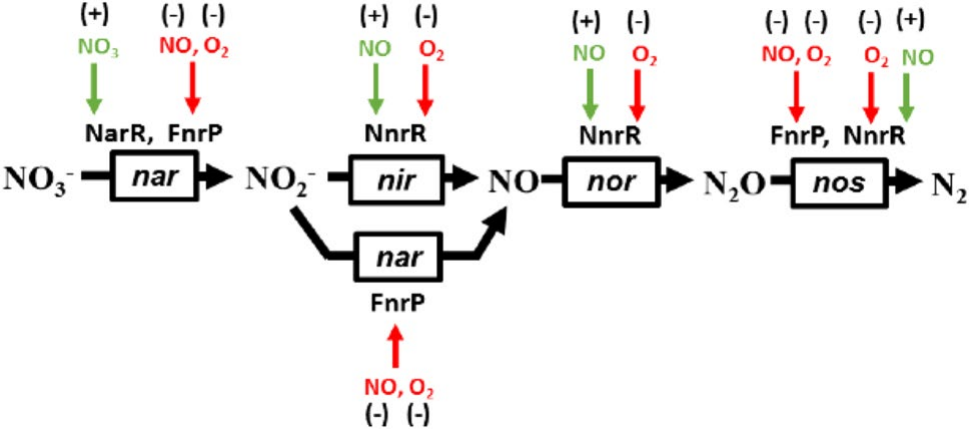
Regulation of denitrification in *P. denitrificans*. Nitrate reductase (*nar*) is co-regulated by NarR and FnrP. Nitrite reductase (*nir,* and nitric oxide reductase (*nor*) are regulated solely by NnrR. Nitrous oxide reductase (*nos*) is co-regulated by FnrP and NnrR. Summarized from references [4, 10, 11, 14]. Small molecule effectors required to activate or inhibit transcription are marked a (+) or (−), respectively. Note: under certain circumstances *nar* will reduce nitrite (NO_2_^−^) to nitric oxide (NO)

FnrP becomes activated under anaerobic conditions by the insertion of an O_2_-labile [4Fe–4S]^2+^ cluster, coordinated by four cysteine residues (Cys14, 17, 25 and 113) present in the N-terminal sensory domain [13]. Cluster incorporation enables the C-terminal DNA-binding domain to recognize specific binding sites located in FnrP regulated promoters. Inactivation of FnrP by O_2_ very likely involves oxidative disassembly of the [4Fe–4S] cluster, causing FnrP to adopt a transcriptionally inactive form [14]. In this way, FnrP regulates most of the genes associated with adaptation to the anaerobic environment, including nitrate reductase (*nar*) and nitrous oxide reductases (*nos*) [4, 10, 11], see Fig. 1. For *E.coli* FNR, exposure to O_2_ drives a [4Fe–4S]^2+^ to [2Fe–2S]^2+^ cluster conversion, causing the FNR dimer to dissociate into transcriptionally inactive monomers [5]. In contrast, FNR orthologues from *P. putida* (ANR) and *B. subtilis* (FnrB) both form a stable dimer under aerobic and anaerobic conditions, independent of the nature of the iron–sulfur cluster [9, 15].

Many bacteria that carry out anaerobic respiration with nitrate/nitrite as a terminal electron acceptor can produce nitric oxide (NO) endogenously [16, 17]. In *P. denitrificans*, NO production represents a key intermediary step during reduction of nitrate to dinitrogen [3]. NO is a gaseous lipophilic radical that can function as both a signaling molecule and a cytotoxic agent [18]. The latter arises from the reactivity of NO with a variety of important cellular targets [19–21], including iron–sulfur cluster proteins [22]. Several regulatory proteins known to respond to NO contain iron–sulfur clusters as the sensory module [5, 23], and significant progress has been made recently in understanding the reactions of iron–sulfur clusters with NO in regulatory proteins [24, 25]. In the case of FnrP, there is growing in vivo evidence to suggest that, in addition to its primary function as an O_2_ sensor, it also plays a role in modulating gene expression in response to NO [10] in a similar way to *E. coli* FNR [25, 26].

Here we report investigations of the biochemical properties of FnrP. We present information on the nature of the iron–sulfur cluster, its reaction with O_2_ and NO, as well as the effect of these reactions on its association state. We compare our findings to those reported for *E. coli* FNR as well other FNR orthologues.

## Materials and methods

### Purification of FnrP

A GST-FnrP fusion protein (Fig. S1) was overproduced in aerobically grown *E. coli* BL21λDE3 harboring pSAD105, induced by the addition of 1 mM IPTG at 37 °C, as previously described [14]. For the in vivo assembly of [4Fe–4S] FnrP, the aeration of cultures harboring pSAD105 was reduced after induction, as previously described [27] to mimic semi-aerobic conditions [11]. FnrP was purified under anaerobic conditions using assay buffer (25 mM HEPES, 2.5 mM CaCl_2_, 100 mM NaCl, 100 mM NaNO_3_, pH 7.5) as previously described for *E. coli* FNR [28]. FnrP was cleaved from the fusion protein using thrombin (Fig. S1), and, where necessary, the [4Fe–4S] cluster reconstituted, in vitro, as previously described [14, 27, 29], except that a 1 ml Q Sepharose column was used to concentrate the protein and assay buffer containing 500 mM KCl was used to elute the protein. Protein concentration was determined using the method of Bradford (BioRad), with bovine serum albumin as the standard [30]. FnrP iron and acid-labile sulfide content were determined as previously described [31, 32].

### Spectroscopy

UV–visible absorbance measurements were made with a Jasco V550 spectrometer. The extinction coefficient for the *E. coli* [4Fe–4S] FNR (*ε*_406 nm_ = 16,200 M^−1^ cm^−1^ [28]) was used to calculate the amount of [4Fe–4S] cluster present in FnrP samples. CD spectra were measured with a Jasco J810 spectropolarimeter. For liquid chromatography–mass spectrometry (LC–MS) an aliquot of FnrP (100 μL, 46 μM [4Fe–4S]) was combined with varying aliquots of aerobic (229 μM O_2_, 20 °C) or anaerobic assay buffer (200 μl final volume), and allowed to react for 15 min. Samples were diluted to ~2 μM final concentration, with an aqueous mixture of 1 % (v/v) acetonitrile, 0.3 % (v/v) formic acid, sealed, removed from the anaerobic cabinet and analyzed by an LC–MS instrument consisting of an Ultimate 3000 UHLPC system (Dionex, Leeds, UK), a ProSwift RP-1S column (4.6 × 50 mm) (Thermo Scientific), and a Bruker microQTOF-QIII mass spectrometer, running Hystar (Bruker Daltonics, Coventry, UK), as previously described [9].

### Gel filtration

FnrP samples (loaded at ~28 μM [4Fe–4S]) before and after exposure to O_2_ were analyzed by gel filtration under anaerobic conditions using assay buffer and a calibrated Sephacryl S-100HR 16/50 column (GE Healthcare), at a flow rate of 1 ml min^−1^.

### Kinetic measurements

Kinetic measurements were performed under pseudo-first-order conditions (162 μM O_2_) at 25 °C by combining varying ratios of aerobic and anaerobic assay buffer (2 ml total volume) with FnrP (8.5 μM [4Fe–4S]). Changes in A_406 nm_ were used to track cluster conversion. A single or double exponential function, as necessary, was fitted to the data, as previously described [9, 28, 33]. Observed rate constants (*k*_obs_) obtained from the fits (in the case of double exponential fits, the rate constant for the first reaction phase was used) were divided by the O_2_ concentration, providing an estimate of the apparent second-order rate constant. Kinetic data fitting was performed using Origin (version 8, Origin Labs). Estimates of errors for rate constants are represented as ±the standard deviation.

### Other analytical methods

FnrP (2 ml) was titrated against varying aliquots of O_2_ (~220 μM dissolved in assay buffer) or NO (as NONOate; Cayman chemicals), using anaerobic cuvettes and gas tight syringes. Stock solutions of the NO donor PROLI-NONOate (*t*½ = 1.5 s) were prepared in 50 mM NaOH and quantified optically (*ε*_252 nm_ 8400 M^−1^ cm^−1^).

## Results and discussion

### In vivo purified and in vitro reconstituted FnrP binds an identical [4Fe–4S] cluster

**Fig. 2:**
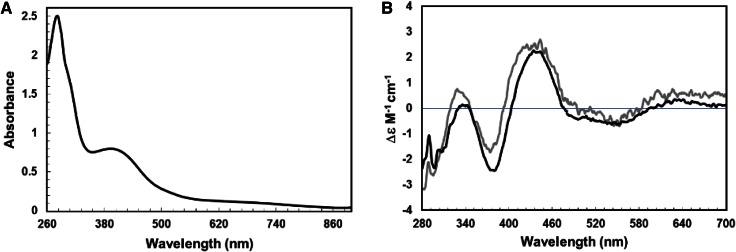
Spectroscopic properties of [4Fe–4S] FnrP. **a**. Absorbance spectrum of reconstituted FnrP (48 μM [4Fe–4S], 81 % cluster loaded), **b** CD spectrum of the same reconstituted FnrP (*black*) and [4Fe–4S] FnrP assembled in vivo (*gray*), for comparison. The buffer was 25 mM HEPES 2.5 mM CaCl_2_ 100 mM NaCl 100 mM NaNO_3_, 500 mM KCl, pH 7.5

Cluster reconstitution of apo-FnrP yielded a straw brown-colored solution with a UV-visible absorbance spectrum (see Fig. 2a) containing a broad shoulder at 420 nm, together with a less well-resolved feature at ~310 nm, indicative of the presence of a [4Fe–4S] cluster, as previously described [14]. A cluster-containing form of FnrP was also expressed in *E. coli* cultures (hereafter referred to as native) and isolated under anaerobic conditions. Reconstituted FnrP samples used in this work contained 0.9 (±0.1) clusters per monomer, based on absorbance measurements, and protein, iron, and acid-labile sulfide determinations. Native samples were found to contain variable amount of the [4Fe–4S] cluster, up to a maximum of ~0.7 per monomer.

As FnrP contains seven cysteine residues (Cys8, 14, 17, 25, 28, 113 and 144), it is possible that differing methods (in vitro versus in vivo) of iron–sulfur cluster assembly might lead to significant differences in the ligation pattern and hence the local environment of the cluster. Since iron–sulfur clusters derive their optical activity from the fold of the protein to which they are ligated, the CD spectrum provides information about the cluster environment. The anaerobic CD spectra of both native and reconstituted [4Fe–4S] FnrP displays positive (+) features at 335 and 440 nm, together with negative (−) features at 300, 375, and 520 nm (see Fig. 2b). The Δ*ε* values for the native and reconstituted forms of FnrP are very similar, indicating that the [4Fe–4S] clusters are in essentially identical environments. Hence, reconstituted FnrP was used in subsequent experiments.

Interestingly, the shape of the CD spectrum of FnrP is quite distinct from that of *E. coli* FNR [28]. The major bands at (−)375 nm and (+)440 nm, in FnrP, which originate primarily from S → Fe charge transitions, are equivalent to bands observed at (+)380 nm and (+)420 nm for [4Fe–4S] FNR and other [4Fe–4S] containing proteins, such as HiPIP, WhiD and ANR [9, 34, 35]. However, there is no strict correlation between the cluster type and the shape or sign of bands in the CD spectrum; presumably, differences in the cluster binding cavity and/or the geometry of the cluster lead to variation in the CD spectrum.

### Reaction of [4Fe–4S] FnrP with O_2_ resembles that of *E. coli* FNR

**Fig. 3:**
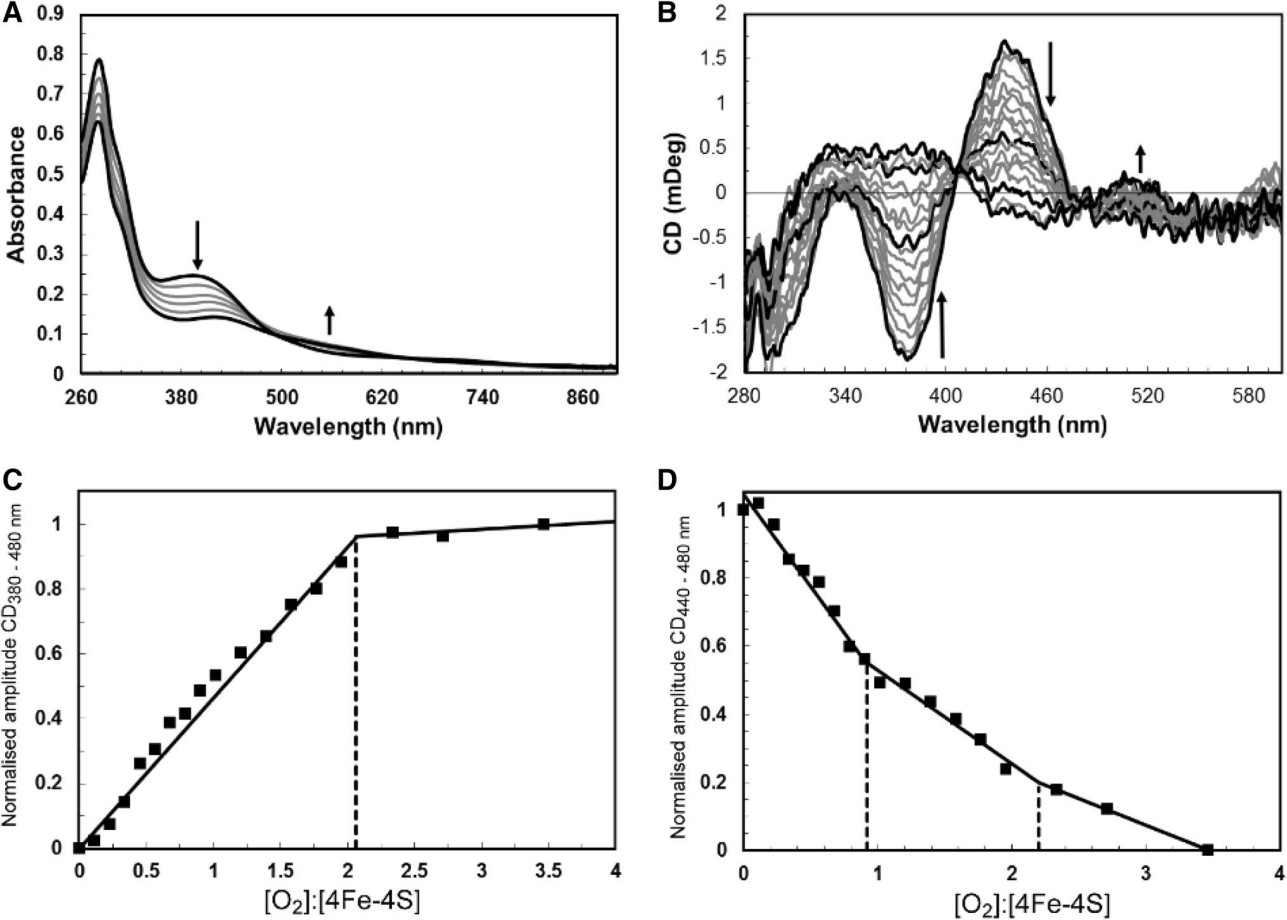
Titration of [4Fe–4S] FnrP with oxygen. **a** Absorbance spectra following addition of O_2_ to [4Fe–4S] FnrP (15 μM in cluster). *Black lines* represent an [O_2_]:[4Fe–4S] ratio of 0 and 5, respectively. **b** CD spectra following addition of O_2_ to [4Fe–4S] FnrP (16 μM in cluster) monitored by circular dichroism. *Black lines* represent [O_2_]:[4Fe–4S] ratios of 0, 1, 2 and 3.5, respectively. *Arrows* indicate the direction of movement in response to O_2_. Spectra recorded at intervening [O_2_]:[4Fe–4S] ratios are shown in *gray*. **c** CD_380–480 nm_ and **d** CD_440–480 nm_ values were normalized and plotted versus the [O_2_]:[4Fe–4S] ratio

Titration of reconstituted FnrP with aerobic buffer (229 μM dissolved O_2_ at 20 °C) revealed a progressive decrease in A_406 nm_ and concomitant increase at 530 nm (see Fig. 3a), features typically associated with an FNR-like [4Fe–4S] to [2Fe–2S] conversion [5, 36]. Clear end points to the titration were not observed at A_406 nm_, possibly due to the slow degradation of the [2Fe–2S] cluster, which contributes to the ΔA_406 nm_ readings. Therefore, an identical titration was followed by CD spectroscopy (see Fig. 3b). The well-resolved features of the [4Fe–4S] cluster were gradually replaced with a broad spectrum reminiscent of *E. coli* [2Fe–2S] FNR, containing positive features at ~360 and ~510 nm, together with a single negative feature at ~450 nm, and isosbestic points at 408 and 480 nm. The spectral features of native [4Fe–4S] FnrP behaved in a comparable manner (not shown) during equivalent titrations. A plot of CD_380 nm_–CD_480 nm_ versus [O_2_]:[4Fe–4S] (Fig. 3c) revealed that the reaction reached completion at an [O_2_]:[4Fe–4S] ratio of ~2; this behavior is notably different to that of *E. coli* FNR [36], which showed that the reaction was 85 % complete at a ratio of 1 [O_2_]:[4Fe–4S]. By contrast, plotting CD_440 nm_–CD_480 nm_ versus [O_2_]:[4Fe–4S] revealed clear inflection points at both 1 and 2 [O_2_]:[4Fe–4S] cluster (see Fig. 3d), suggesting the presence of a meta-stable intermediate.

**Fig. 4:**
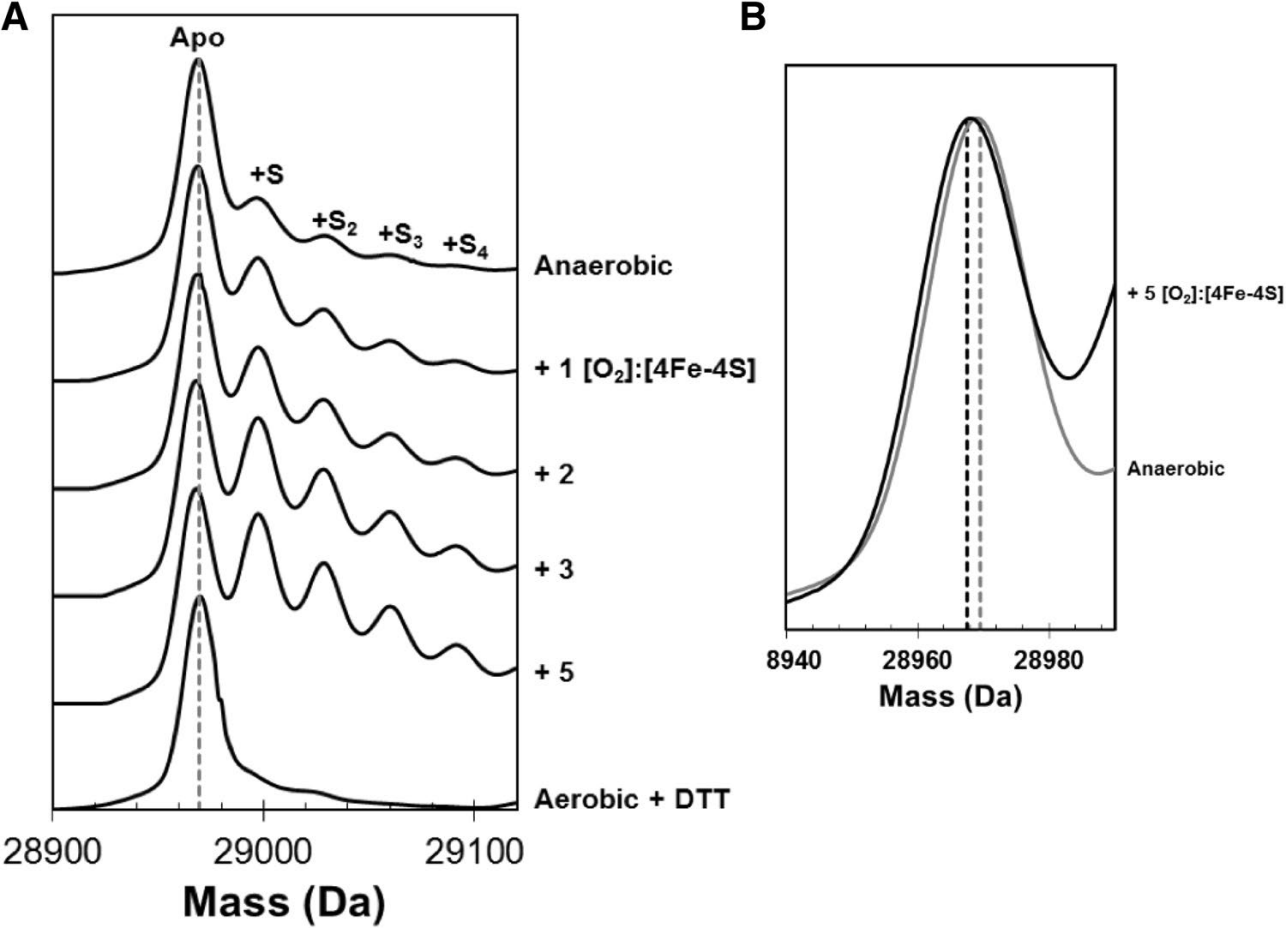
Detection of persulfide species of FnrP by LC–MS**. a** ESI-TOF mass spectra of [4Fe–4S] FnrP (2.3 μM) before and after the addition of increasing amounts of O_2_ are shown, as indicated. The peak at 28,969 Da corresponds to the monomer molecular ion peak of FnrP, and the peaks at +32, +64, +96 and +128 Da correspond to the addition of one, two, three and four covalently bound sulfur atoms, respectively, as indicated. Treatment of an aerobic ([O_2_]:[4Fe–4S] = 5) sample, post-O_2_ exposure, with DTT led to loss of persulfide adducts. **b** During the titration, the mass of the FnrP decreased from 28,969 to 28,967 Da, corresponding to the loss of 2 protons and indicative of O_2_-induced disulfide bond formation

Analysis of iron–sulfur proteins via liquid chromatography–mass spectroscopy (LC–MS) invariably leads to the loss of the cluster, although previous studies have shown that cysteine persulfides formed during the reaction of the cluster with O_2_ remain intact [37]. It was, therefore, of interest to determine if O_2_-mediated cluster conversion in FnrP resulted in retention of cluster sulfides as covalent adducts. [4Fe–4S] FnrP was treated with increasing amounts of O_2_ (up to 5 molar equivalents) for 15 min, and analyzed by LC–MS, see Fig. 4a. Even prior to the introduction of O_2_, a small amount of sulfur adduct (at +32 Da of the 28,969 Da peak) was observed. As O_2_ was introduced, the relative abundance of these adducts increased, with 1–4 persulfide adducts observed and with the single persulfide adduct at +32 Da the most abundant. Furthermore, the mass of the main protein peak decreased by 1–2 Da, indicating formation of disulfide bonds (Fig. 4b). Treatment with 2 mM DTT, post-O_2_ exposure, resulted in the loss of all sulfur adducts and reduction of disulfides.

**Scheme 1:**
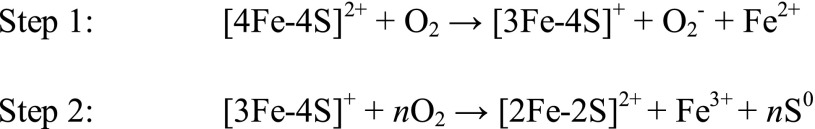
Reaction of E. coli [4Fe-4S] FNR with O_2_

The [4Fe–4S] cluster of *E. coli* FNR has been shown to react with O_2_ via a two-step mechanism (see Scheme 1). Step 1 involves the one electron oxidation of the [4Fe–4S]^2+^ cluster leading to release of Fe^2+^ to generate a [3Fe–4S]^1+^ intermediate along with superoxide, which may be recycled back to O_2_ by the combined actions of superoxide dismutase (SOD) and catalase [33]. Step 2 involves the conversion of the [3Fe–4S]^+^ cluster to the [2Fe–2S]^2+^ cluster, with the concomitant release of a further iron ion and oxidation of cluster sulfides, which can be stored as cysteine persulfide [5, 33, 37]. In the case of FnrP, the two inflection points observed in the CD intensity plot (Fig. 3d) together with the LC–MS data (Fig. 4) indicate that the availability of O_2_ beyond a ratio of 1 per cluster results in increased sulfide oxidation/persulfide formation. This, causes further changes in the CD spectrum, up to a ratio of 2 [O_2_]:[4Fe–4S], with little further effect beyond this, suggesting that persulfidation of cluster-coordinating Cys residues occurs mostly in the 1–2 [O_2_]:[4Fe–4S] range.

### FnrP reacts more slowly than *E. coli* FNR with O_2_ in vitro

**Fig. 5:**
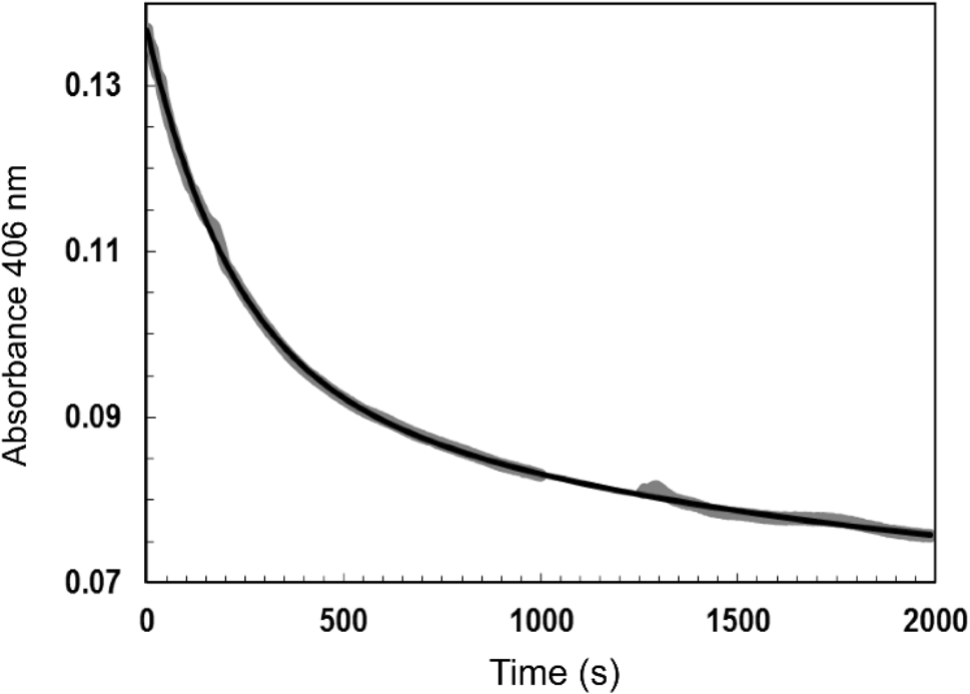
Kinetics of O_2_-mediated [4Fe–4S] cluster conversion. Kinetics were recorded under pseudo-first-order conditions (8.5 μM [4Fe–4S], 161.6 μM O_2_) at an [O_2_]:[4Fe–4S] ratio of 19 at 25 °C. A double exponential function (*black* *line*) was used to fit the data (*gray*). The rate constants reported in the text from these experiments are mean values with standard errors from two repeats

The first clue that [4Fe–4S] FnrP may be less sensitive to O_2_ was the isolation of a straw brown protein from semi-aerobic cultures. Equivalent preparations with *E. coli* FNR typically yield apo-FNR or occasionally [2Fe–2S] FNR [38, 39]; anaerobic cultures are required to obtain [4Fe–4S] FNR [27, 40]. Hence, it was of interest to investigate the kinetics of O_2_-induced [4Fe–4S] FnrP cluster conversion. The A_406 nm_ decay for FnrP was measured under pseudo-first-order reaction conditions ([O_2_]:[4Fe–4S] ratio of ~19). The data were fitted best by a double exponential function (consistent with a two-step reaction [33]) with an observed rate constant (*k*_obs_) for the first reaction of 0.0047 (±0.0001) s^−1^ (see Fig. 5). Division of *k*_obs_ by the O_2_ concentration (161 μM) provides an estimate of the apparent second-order rate constant, *k* = 29 (±1) M^−1^ s^−1^. This value establishes that the [4Fe–4S] cluster of FnrP is significantly less reactive with O_2_ in vitro than the archetypal *E. coli* FNR cluster [28], and that it more closely resembles the *Pseudomonas putida* FNR proteins PP_3233 and PP_3287 and the previously characterized variant of *E.coli* FNR, FNR-S24F, in its reactivity (see Table 1) [9, 41].

**Table 1:** Kinetic data for the reaction of FNR homologues with O_2_

Species	Protein ID	Second-order rate constant (M^−1^ s^−1^)	References
*E. coli*	FNR	180–200	[5]
	FNR-D154A	167–172	[5]
	FNR-I151A	130	[5]
	FNR-S24F	80	[41]
*N. meningitidis*	NmFNR	105	[55]
*P. putida*	ANR	280	[9]
	PP_3287	55	[9]
	PP_3233	38	[9]
*P. denitrificans*	FnrP	29	This study

**Fig. 6:**
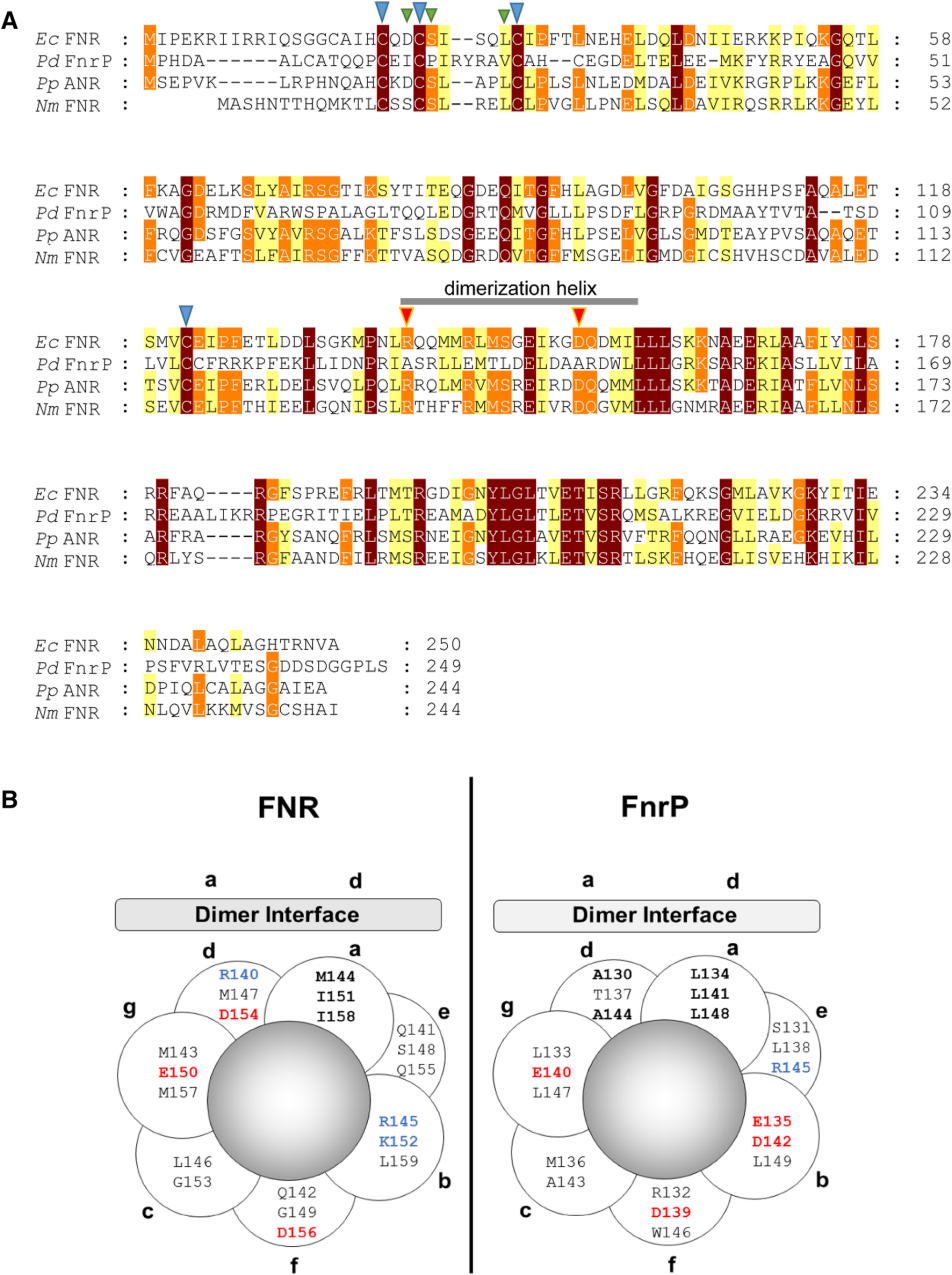
Sequence alignments of FNR proteins and comparison of the dimerization helixes of FNR and FnrP. **a** Sequence alignment of FNR proteins. The dimerization helix is indicated. Cluster-coordinating residues are indicated by *blue* *arrowheads*. Residues next to cluster-coordinating Cys residues that are important for controlling cluster reactivity are indicated by *green* *arrowheads*. Two key residues within the helix that are important for *E. coli* FNR association state, Arg140 and Asp154, are indicated by *orange* *arrowheads*. Proteins are *E. coli* FNR (*Ec*FNR), *Paracoccus denitrificans* FnrP (*Pd*FnrP), *Pseudomonas aeruginosa* FNR (*Pa*NnrR) and *Neisseria meningitidis* FNR (*Nm*CRP). The alignment was generated using Clustal Omega [53] and annotated using Genedoc [54]. **b** Helical wheel projection of the dimerization helix of **a** *E. coli* FNR and **b** *P. denitrificans* FnrP, assuming the standard 3.5 residues per turn for a coiled coil. Residues that when substituted by alanine result in a significantly altered activity in *E. coli* FNR, and predicted equivalents in FnrP, occupy positions *a* and *d* of the helical wheel. Adapted from Moore et al. [46]

Previous work with *E. coli* FNR showed that replacement of Ser24, located immediately adjacent to the cluster ligand Cys23, by Pro results in significant aerobic FNR activity, indicative of FeS cluster stabilization [41]. Interestingly, FnrP has Pro in the position equivalent to Ser24 in FNR (Fig. 6a). Thus, this amino acid residue substitution could, at least partially, account for the lower O_2_ reactivity. We note that amino acid substitutions at other positions next to cluster-coordinating Cys residues are also known to influence the aerobic reactivity of the *E. coli* FNR cluster with O_2_. For example, substitutions of Asp22 with Ser or Gly resulted in increased activity under aerobic conditions [42, 43]. The equivalent position in FnrP is occupied by Ile (Fig. 6a) and this difference likely alters the redox properties of the cluster, resulting in a lower reactivity towards O_2_.

Under O_2_-limiting conditions many bacteria induce high-affinity oxidases, as the ATP yield from oxygen respiration is significantly higher than anaerobic respiration [2]. *P. denitrificans* is no different in this respect, with FnrP activating the expression of the *cbb*_3_-type cytochrome *c* oxidase (*cco*) in vivo [2, 11]. We note that *cco* promoter activity increases by eight times during the switch from aerobic to semi-aerobic growth conditions [11]. In contrast, the high-affinity *E. coli* cytochrome *bd*-I (*cyd*AB) oxidase (a quinol:O_2_ oxidoreductase) is repressed by [4Fe–4S] FNR under anaerobic conditions [44, 45]. Thus, *P. denitrificans* may begin to utilize its high-affinity oxidases at significantly higher environmental O_2_ concentrations than its rivals to gain a competitive advantage.

### The oligomeric state of FnrP is dependent on the [4Fe–4S] cluster

**Fig. 7:**
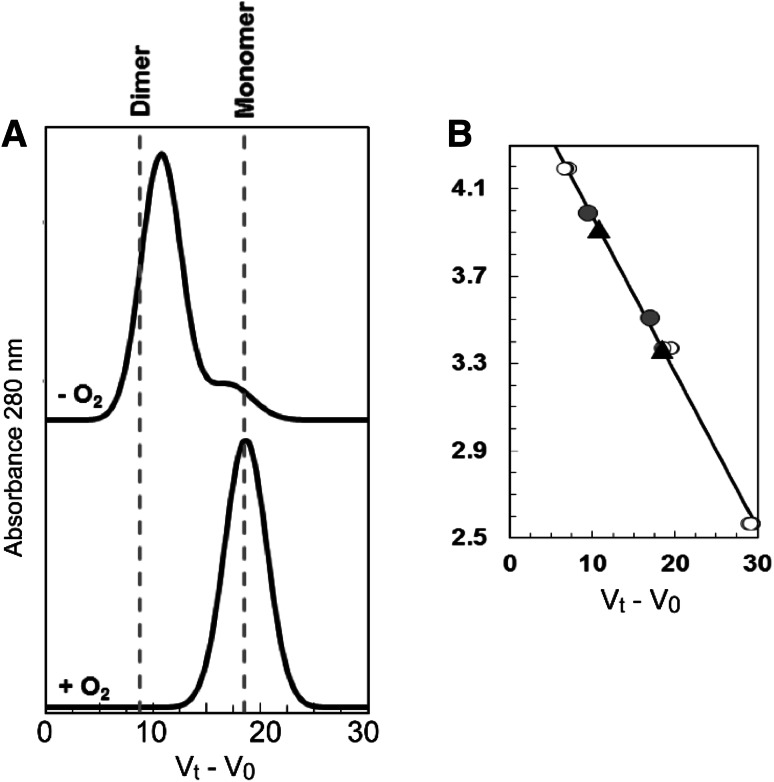
Association state properties of FnrP probed by gel filtration. **a** Chromatogram for reconstituted [4Fe–4S] FnrP in the absence (*upper trace*) or presence of O_2_ (*lower trace*). In the absence of O_2_ FnrP had an apparent molecular mass of ~51 kDa, indicative of a dimer. A small shoulder, corresponding to a mass of ~32 kDa (monomer) is also observed. In the presence of O_2_, FnrP had an apparent mass of ~29 kDa, consistent with monomeric protein. **b** Standard calibration curve for a Sephacryl S100HR column. *Open circles* correspond to standard proteins (BSA, carbonic anhydrase, cytochrome *c*), *gray circles* correspond to the two states of *E. coli* FNR. *Black triangles* correspond to the two states of FnrP. Ln *M*_w_, natural log of molecular weight in kDa

Under anaerobic conditions *E. coli* FNR acquires a [4Fe–4S] cluster, triggering a conformational change at the dimerization interface that leads to the formation of homo-dimers (~60 kDa) and site-specific DNA binding [5]. Upon exposure to O_2_, a [4Fe–4S] to [2Fe–2S] cluster conversion results in a rearrangement of the dimer interface, leading to monomerization (~30 kDa) [46]. To date, only *E. coli* FNR has been reported to undergo this monomer/dimer transition; with other FNR orthologues tending to remain dimeric irrespective of the cluster [9, 15]. Therefore, the oligomeric state of reconstituted [4Fe–4S] FnrP (~90 % loaded) was examined by analytical gel filtration (see Fig. 7). FnrP has a mass of ~29 kDa, per monomer. In the absence of O_2_, the majority of the protein eluted with a relative molecular mass of 51.0 (±1.0) kDa, somewhat lower than, but close to, the expected mass for a homodimer. We note that a slight shoulder, corresponding to a relative molecular mass of ~32 kDa, was also observed. In the presence of O_2_, the protein eluted with a relative molecular mass of 28.8 (±0.2) kDa, as expected for monomeric FnrP. These results indicate that cluster conversion drives a rearrangement of the dimer interface, leading to monomerization, as observed for *E. coli* FNR.

Moore and Kiley [46] showed that subunit interactions in *E. coli* FNR arise from a predominantly hydrophobic interface (see Fig. 6b), and that the negatively charged side chain of Asp154 is oriented towards this interface, where inter-subunit charge repulsion inhibits dimerization before cluster acquisition. Insertion of the [4Fe–4S]^2+^ cluster apparently causes shielding of the negative charge, by Ile-151, thereby facilitating dimerization. The recent structure of the *E. coli* FNR-like FNR from *Aliivibrio fischeri* [47] was broadly in agreement with this model, but indicated that hydrophobic contacts made between Ile-151 residues of the two subunits, rather than screening electrostatic repulsion, is key to stabilizing the dimer. Removal of the negatively charged side chain by substitution, such as in FNR-D154A, alleviates the repulsion even in the absence of a cluster, leading to a predominantly dimeric form whether the cluster is present or not. The positive charge of Arg140 is also important for FNR function. Substitution of this positively charged side chain, such as in FNR-R140A, resulted in an FNR variant with little anaerobic activity, implying a defect in dimerization. In the *A. fischeri* FNR structure, Arg-140 is located near the N terminus of the dimerization helix where no dimerization helix interactions occur. Instead, the Arg-140 sidechain forms a salt bridge with Asp-130 of the other subunit (B helix) and it was suggested that this interaction plays a key role in dimer stability. Interestingly, the double mutant FNR-R140A/D154A regained ≥70 % activity under anaerobic conditions [46]. Assuming residues 130 to 149 participate in subunit interactions, analogous to *E. coli* FNR residues 140 to 159 (Fig. 6a), then Leu134, 141 and 148 presumably form the core of the hydrophobic interface in FnrP (see Fig. 6b). Moreover, charged residues Arg140 and Asp154, found to be important to the monomer/dimer equilibrium in *E. coli* FNR, appear to be replaced by Ala130 and Ala144 in FnrP. Thus, the ability of FnrP to undergo a cluster-induced monomer/dimer transition may depend on the lack of strong electrostatic interactions involving the side chains of Ala130 and Ala144 (*cf* the *E. coli* FNR variant R140A/D154A [46]). The remainder of the residues in FnrP appears to preserve the general nature of the *E. coli* FNR dimerization helix (see Fig. 6). We note that *Bradyrhizobium japonicum* FixK2 also contains Ala residues at equivalent positions to FnrP Ala130 and 144, and that other CRP/FNR family paralogues tend to contain an Ala residue in place of the Arg residue and a hydrophobic/non-charged residue in place of the negatively charged Asp residue (see Fig. S2).

### FnrP is a nitric oxide (NO) sensor

**Fig. 8:**
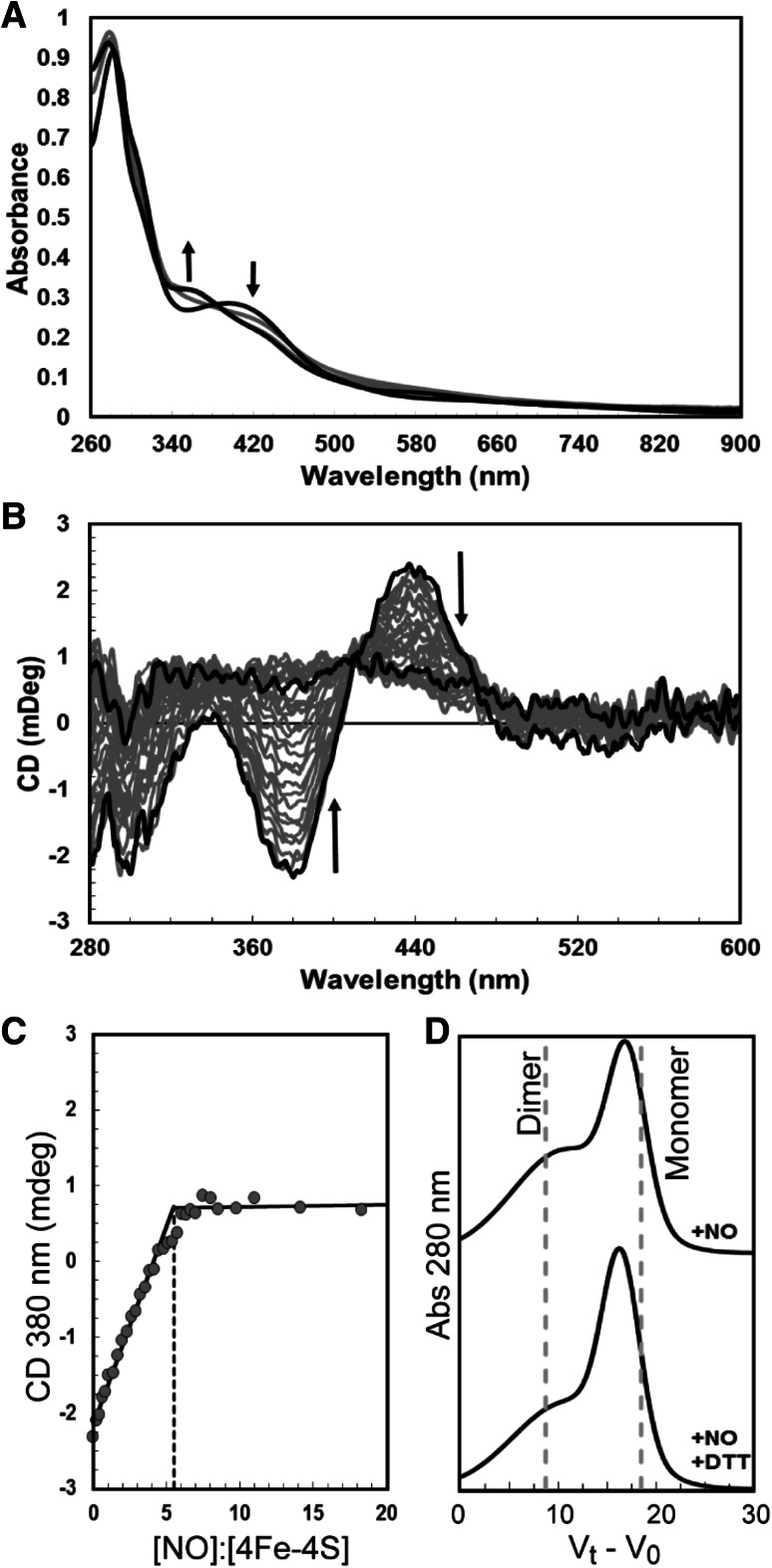
Titration of [4Fe–4S] FnrP with NO. **a** Absorbance spectra of [4Fe–4S] FnrP (18 μM [4Fe–4S]) following exposure to NO. After each addition of Proli-NONOate, the sample was incubated at an ambient temperature for 5 min prior to spectroscopic measurements. *Black lines* correspond to [NO]:[4Fe–4S] ratios of 0 and 18. Intervening spectra (*gray lines*) correspond to ratios of 4 and 8. **b** CD spectra of an equivalent titration (18 μM [4Fe–4S]). *Black lines* correspond to [NO]:[4Fe–4S] ratios of 0 and 18, intervening spectra are *gray*. **c** CD_380 nm_ values were normalized and plotted versus the [NO]:[4Fe–4S] ratio. **d** Gel filtration analysis of nitrosylated FnrP. In the presence of NO, FnrP had a molecular mass of ~33 kDa (monomer). A broad shoulder, corresponding to a mass of 50–100 kDa, was also observed. The shape of this shoulder decreased in the presence of DTT, implying that it may arise from a disulfide-bonded FnrP aggregate

In *P. denitrificans* at least three FNR-like transcriptional regulators are involved in the regulation of the four denitrification reductases (see Fig. 1), in response to nitrate, NO or O_2_ [4]. In vivo studies have shown that FnrP is involved in the dual control of both the nitrate reductase (*nar*) and the nitrous oxide reductase *(nos)* with NarR and NnrR, respectively, and that factors besides O_2_ influence the activity of FnrP [10, 12, 48]. In this respect, we note that the *cco* promoter activity drops to one-third of the semi-aerobic level when cultures are shifted from semi-aerobic to denitrifying conditions and that cultures experience a transitory accumulation of NO under anaerobic conditions prior to the establishment of denitrification. There is also increasing evidence to suggest that FnrP, like *E. coli* FNR, also responds to NO in vivo [10, 11]. Therefore, we investigated the stoichiometry of the reaction of the FnrP cluster with NO by measuring changes in CD and absorbance spectral properties following sequential additions of NO to anaerobic FnrP. A progressive decrease in A_406 nm_ and an increase in A_360 nm_ was observed. The final UV–visible spectrum, with a principal absorption band at 360 nm and a shoulder at 430 nm are consistent with previous observations of reaction of an iron–sulfur cluster with NO and indicate the formation of iron–nitrosyl species (see Fig. 8a) [24, 25]. CD signals arising from the [4Fe–4S] cluster decreased almost to zero as the reaction with NO proceeded (Fig. 8b). A plot of intensity at 380 or 430 nm versus [NO]:[4Fe–4S] (Fig. 8C) revealed that the reaction was complete at a stoichiometry of ≥7 NO molecules per cluster, with a clear inflection point at ~6 NO, similar to the previously reported nitrosylation of *E. coli* FNR [25].

Analysis by gel filtration of molecular mass changes upon nitrosylation of [4Fe–4S] FnrP revealed a decrease in mass from 51 to 33 kDa (Fig. 8d) for the bulk of the sample. A broad shoulder, corresponding to a mass of ~50–100 kDa, was also observed. The shoulder decreased following treatment with DTT, implying that a small proportion of the sample was in the form of a disulfide-bonded FnrP aggregate. Moreover, LC–MS analysis of nitrosylated FnrP revealed the presence of sulfur adducts (not shown), as previously observed for *E. coli* FNR [25]. We conclude that nitrosylation of FnrP is likely to proceed in a manner similar to that previously reported for *E. coli* FNR, and that, like its reaction with O_2_, nitrosylation of FnrP results in dissociation of the dimer into monomers, again similar to *E. coli* FNR.

## Conclusion

FNR proteins are global transcription factors that respond to changes in environmental O_2_ through the assembly and disassembly of an O_2_-sensitive [4Fe–4S] cluster. In the archetypal FNR protein *E. coli* FNR, molecular O_2_ brings about conversion of the [4Fe–4S] cluster into a [2Fe–2S] form, thereby triggering conformational changes that initiate monomerization and concomitant loss of sequence-specific DNA binding. In this respect, the *P. denitrificans* regulator FnrP is similar to its *E. coli* counterpart [5]. However, sequence alignment revealed that the residues important for the monomer–dimer equilibrium for FnrP are different to those in *E. coli* FNR and involve fewer charged side chain interactions. Furthermore, we have found that the FnrP cluster is at least 6 times less sensitive to O_2_ than *E. coli* FNR. This finding is consistent with the observation of FnrP-activated expression of *cbb*_3_-type cytochrome *c* oxidase, nitrate reductase and nitrous oxide reductase under semi-aerobic conditions in vivo [11, 48].

Many transcriptional regulators are known to respond to NO and in *P. denitrificans* the principal regulators are NsrR, NnrR, and FnrP. In other bacterial species NsrR regulates, amongst others, genes involved in detoxification, such as *hmp*, for which NO is a substrate [49, 50]. NnrR principally activates the expression of the nitrite, nitric oxide, and nitrous oxide reductases under anaerobic conditions in response to NO, thereby facilitating denitrification [13, 51, 52]. With regard to denitrification, FnrP co-regulates the expression of the nitrate and nitrous oxide reductases, ensuring their production under semi-aerobic conditions. We note that nitrate reductase is the most important source of endogenously derived NO during nitrate/nitrite respiration [16]. It is suggested that if the NO detoxification systems are overwhelmed, FnrP will become nitrosylated leading to lowered expression of *cco*, and modulation of the *nar* and *nos* operons (that require [4Fe–4S] FnrP for activation). The concomitant detection of NO by NnrR would then ensure the timely expression of the *nir*, *nor* and *nos* operons, minimizing the transitory nitrosative stress as metabolic modes are switched over in favor of denitrification. Fig. S3 shows a summary of these regulatory systems. Here we have shown that [4Fe–4S] FnrP undergoes a nitrosylation reaction involving multiple NO molecules. This leads to dissociation of FnrP, containing iron–nitrosyl products similar to those observed for other NO-sensing iron–sulfur regulatory proteins, into monomers, providing a mechanistic basis for NO regulation of FnrP.

## Electronic supplementary material

Supplementary material 1 (PDF 490 kb)

## Abbreviations

DTT: Dithiothreitol
GST: Glutathione S-transferase
FNR: Fumarate nitrate reduction
HEPES: 4-(2-Hydroxyethyl)-1-piperazine-ethanesulfonic acid
IPTG: Isopropyl β-d-1-thiogalactopyranoside
LC–MS: Liquid chromatography mass spectrometry
NO: Nitric oxide
PROLI-NONOate: 1-(Hydroxy-NNO-azoxy)-l-proline, disodium salt
SOD: Superoxide dismutase
