# Supplementary material for: Biochemical properties of Paracoccus denitrificans FnrP: reactions with molecular oxygen and nitric oxide
Source: J Biol Inorg Chem. 2016 Jan 20;21:71–82. doi: 10.1007/s00775-015-1326-7 (PMC4771820; doi:10.1007/s00775-015-1326-7)
Supplement: Supplementary file 1 — Supplementary material 1 (PDF 490 kb) [file 775_2015_1326_MOESM1_ESM.pdf]

Supplementary Material

Biochemical properties of *Paracoccus denitrificans* FnrP: Reactions with molecular oxygen and nitric oxide

Jason C. Crack, Matthew I. Hutchings, Andrew J. Thomson, and Nick E. Le Brun

Fusion protein

```
MSPILGYWKIKGLVQPTRLLLEYLEEKYEEHLYERDEGDKWRNKKFELGLEFPNLPYYIDGDVKLTQSMAIIRYI
ADKHNMLGGCPKERAEISMLEGAVLDIRYGVSRIAYSKDFETLKVDFLSKLPEMLKMFEDRLCHKTYLNGDHVTH
PDFMLYDALDVVLYMDPMCLDAFPKLVCFKKRIEAIPQIDKYLKSSKYIAWPLQGWQATFGGGDHPPKSDLVPRG
SPGISGGGGGILDSMAHDAALCATQQPCEICPIRYRAVCAHCEGDELTELEEMKFYRRYEAGQVVVWAGDRMDFV
ASVVAGMAGLTQQLEDGRTQMVGLLLPSDFLGRPGRDMAAYTVTATSDLVLCCFRRKPFEKLLIDNPRIASRLLE
MTLDELDAARDWLLLLGRKSAREKIASLLVILARREAALIKRRPEGRITIELPLTREAMADYLGLTLETVSRQMS
ALKREGVIELDGKRRVIVPSFVRLVTESGDDSDGGPLS
```

Expected mass: 55117.9

GST remnant

```
MSPILGYWKIKGLVQPTRLLLEYLEEKYEEHLYERDEGDKWRNKKFELGLEFPNLPYYIDGDVKLTQSMAIIRYI
ADKHNMLGGCPKERAEISMLEGAVLDIRYGVSRIAYSKDFETLKVDFLSKLPEMLKMFEDRLCHKTYLNGDHVTH
PDFMLYDALDVVLYMDPMCLDAFPKLVCFKKRIEAIPQIDKYLKSSKYIAWPLQGWQATFGGGDHPPKSDLVPR
```

Expected Mass: 26166.4

Linker FnrP

```
GSPGISGGGGGILDSMAHDAALCATQQPCEICPIRYRAVCAHCEGDELTELEEMKFYRRYEAGQVVVWAGDRMDF
VASVVAGMAGLTQQLEDGRTQMVGLLLPSDFLGRPGRDMAAYTVTATSDLVLCCFRRKPFEKLLIDNPRIASRLL
EMTLDELDAARDWLLLLGRKSAREKIASLLVILARREAALIKRRPEGRITIELPLTREAMADYLGLTLETVSRQM
SALKREGVIELDGKRRVIVPSFVRLVTESGDDSDGGPLS
```

Expected Mass: 28969.5

Fig. S1. Sequence information and predicted molecular masses for GST-FnrP and thrombin derived cleavage products. The N-terminal Cys-rich region of FnrP occurs between the highlighted Met residues, and Cys residues are highlighted in yellow in the cleaved sequence. The released apo-FnrP species had a predicted mass of 28,969.5 Da.

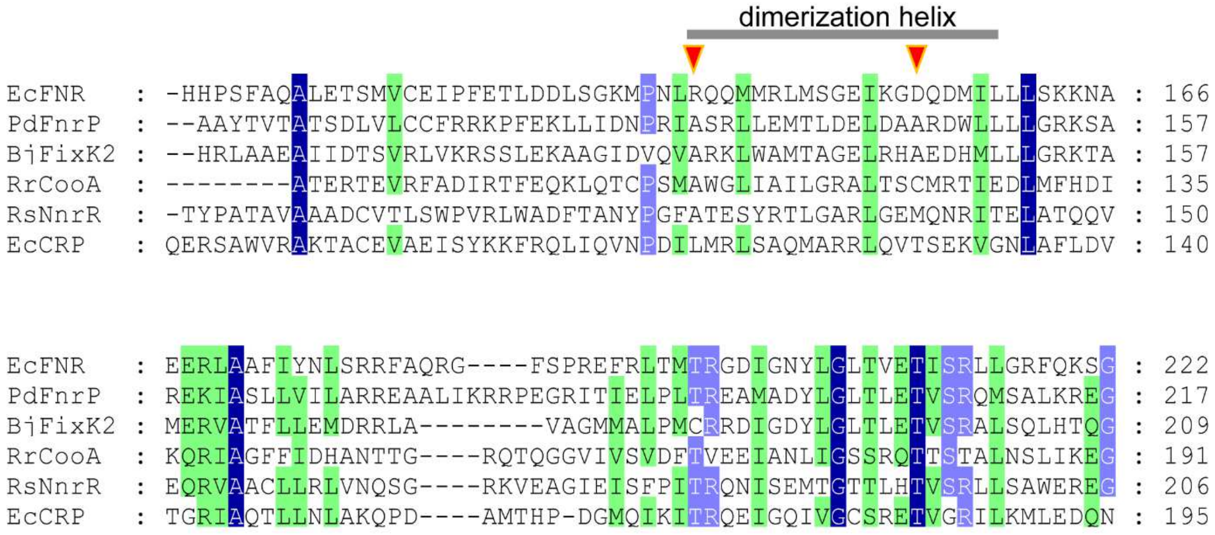

Fig. S2. Partial sequence alignment of CRP/FNR family proteins. The dimerization helix is indicated. Two key residues within the helix that are important for *E. coli* FNR association state, Arg140 and Asp154, are indicated by orange arrowheads. Proteins are *E. coli* FNR (EcFNR), *Paracoccus denitrificans* FnrP (PdFnrP), *Bradyrhizobium japonicum* FixK2 (BjFixK2), *Rhodospirillum rubrum* CooA (RrCooA), *Rhodobacter sphaeroides* NnrR (RsNnrR) and *E. coli* CRP (EcCRP). The alignment was generated using Clustal Omega [1] and annotated using Genedoc [2].

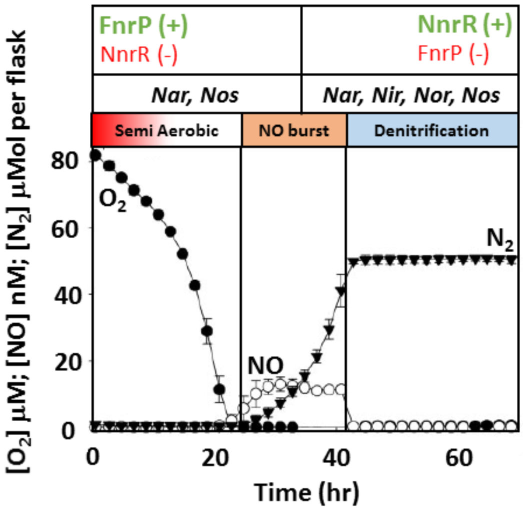

Fig. S3. Temporal regulation of denitrification by FnrP and NnrR in *P. denitrificans*. Under semi-aerobic conditions FnrP activates the expression of nitrate (*nar)* and nitrous oxide (*nos) reductases* [3]. Endogenously derived NO during nitrate/nitrate respiration causes a transient burst of NO production under anaerobic conditions, leading to nitrosylation of FnrP. Concomitant activation of NnrR by NO allows the timely expression of nitrite (*nir*), nitric oxide (*nor*) reductase, completing the denitrification metabolic pathway. The levels of O_2, NO and N_2 are those found in the head space of the culture. Adapted from Bergaust *et al* [3].

References

1. Sievers F, Wilm A, Dineen D, Gibson TJ, Karplus K, Li W, Lopez R, McWilliam H, Remmert M, Soding J, Thompson JD, Higgins DG (2011) Fast, scalable generation of high-quality protein multiple sequence alignments using Clustal Omega. Mol Sys Biol 7:539
2. Nicholas KB, Nicholas HBJ (1997) Genedoc: A tool for editing and annotating multiple sequence alignments, *Distributed by the authors*.
3. Bergaust L, van Spanning RJ, Frostegard A, Bakken LR (2012) Expression of nitrous oxide reductase in Paracoccus denitrificans is regulated by oxygen and nitric oxide through FnrP and NNR. Microbiology 158:826-834.
